# Brief Report of a New Anatomical Region at Risk in Thoracic Radiotherapy: From Discovery to Implementation

**DOI:** 10.1016/j.jtocrr.2024.100742

**Published:** 2024-10-18

**Authors:** Kathryn Banfill, Thomas Marchant, Alan McWilliam, Joseph Wood, Matthias Schmitt, Azadeh Abravan, Gareth Price, Marcel van Herk, Corinne Faivre-Finn

**Affiliations:** a The Christie NHS Foundation Trust, Manchester, United Kingdom; b Radiotherapy Related Research, Department of Cancer Sciences, The University of Manchester, Manchester, United Kingdom; c The North West Heart Centre, Wythenshawe Hospital, Manchester Foundation NHS Trust, Manchester, United Kingdom

**Keywords:** Lung cancer, Radiotherapy, Cardiac toxicity, Organ at risk

## Abstract

Increasing radiotherapy dose to select cardiac structures is associated with cardiac events and premature death. Previous studies have found a dose–response relationship for structures at the base of the heart.

We have defined a new cardiac anatomical region at risk for radiotherapy by consensus opinion, based on image-based data-mining studies. The cardiac avoidance area comprises the superior vena cava, right atrium, aortic root, left main coronary artery, and proximal segments of the left anterior descending and right coronary arteries. We describe a contouring atlas for the cardiac avoidance area to facilitate implementation.

## Introduction

The last 10 years have seen improved survival for patients with lung cancer through better radiotherapy and surgical techniques in addition to the use of immunotherapy in locally advanced disease. There is now a need to consider prevention and management of treatment toxicity to further improve patients’ survival and quality of life.

Radiotherapy dose to the heart in patients treated for lung cancer is associated with poorer survival and increased cardiac events.^1^ Unlike in breast cancer, where dose is delivered to the anterior heart and left anterior descending (LAD) coronary artery, the dose received by the heart in patients with lung cancer depends on tumor stage and location. Whole heart dose parameters do not consider the complexity of the heart’s inter-related substructures with different functions and potentially different dose–responses. We summarize the evidence for avoiding the base of the heart in thoracic radiotherapy, describe for the first time a new cardiac anatomical region at risk, and reveal how we have implemented an avoidance strategy in the routine setting for patients with lung cancer.

## Discovery of a New Anatomical Region at Risk

A number of studies using image-based data-mining (IBDM) have identified an association between radiation dose to the base of the heart and both survival and cardiac events.^2^, ^3^, ^4^ This work used three-dimensional planned radiotherapy dose distributions from hundreds of patients to correlate dose with survival. The dose distributions were deformably registered to a reference patient, and then a *t* test was performed comparing the dose at each voxel with survival. Permutation testing was carried out to account for multiple comparisons and to define an anatomical region where excess dose is significantly associated with survival or cardiac events (*p* < 0.001).^5^

**Figure 1:**
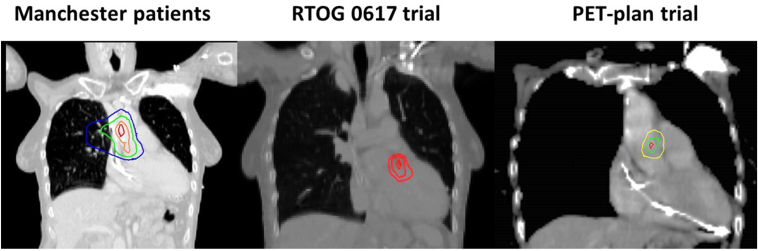
Dose-sensitive cardiac subregions found on image-based data-mining from retrospective study of patients treated at The Christie, RTOG 0617 trial, and PET-plan trial.

IBDM defined anatomical regions where dose was significantly correlated with survival, without the need for prior assumptions and structure delineation, allowing the identification of radiosensitive subregions within an organ. These findings were validated in two external clinical trial data sets, RTOG 0617^2^ and PET-Plan.^4^ Figure 1 reveals the dose-sensitive region in each of these studies.

In addition to IBDM, real-world data analyses from other sources have revealed correlations between the cardiac atria or superior vena cava (SVC), survival, and cardiac events. These studies are summarized in Table 1.

**Table 1:** Thoracic Radiotherapy Studies Revealing the Relationship Between Dose to the Base of the Heart and Outcome

Trial	Data Source	Substructure Identification Method	End Point	Patients Included in Analysis and Stage	Significant Cardiac Substructures	Radiotherapy Planning Modality
McWilliam et al. 2017^3^	Retrospective single-center study of 1163 patients with lung cancer treated with 55 Gy in 20 fractions from 2010 to 2013	IBDM	Overall survival	457 Stage 1 136 Stage 2 508 Stage 3	Base of heart dose correlated with poorer survival	296 IMRT 805 3D-RT
Craddock et al. 2023^4^	205 patients in PET Plan trial	IBDM	Overall survival	172 Inoperable stage 2 or 3 NSCLC	Base of heart dose associated with poorer survival. Larger effect in patients with lower baseline ejection fraction	111 IMRT 93 3D-RT 1 Unknown
McWilliam et al.^2^	490 patients in RTOG 0617 trial	IBDM	Overall survival	458 Inoperable stage 3 NSCLC	Base of heart dose associated with poorer survival	244 3D-RT 214 IMRT
Stam et al. 2017^15^	1337 patients treated with SABR at 5 international centers	Non-rigid registration to average anatomy then substructures contoured on average anatomy	Non-cancer death	803 Patients with stage 1 NSCLC	Near minimum dose to SVC and maximum dose to left atrium associated with noncancer death	Not available
Hotca et al. 2019^16^	Retrospective single-center study of 241 patients treated with 50–80 Gy in 1.8–2 Gy fractions 2004–2014	Atlas based contouring, adapted by user	New ECG changes	155 Inoperable stage 3 NSCLC	Higher minimum dose to SVC associated with new nonspecific ECG changes	155 IMRT
Vivekanandan et al. 2017^17^	82 Patients in IDEAL-CRT trial	Substructures delineated by clinical oncologist	Overall survival ECG changes	6 Stage 2 NSCLC 72 Stage 3 NSCLC	Left atrial wall V63Gy > 2.2% associated with poorer survival No association between substructure doses and ECG change	3 IMRT 79 3D-RT
McWilliam et al. 2020^7^	Retrospective single-center study of 1161 patients with NSCLC 2010–2016	14 Cardiac substructures delineated on 5 template patients. All patients nonrigidly registered to template patient	Overall survival	457 Stage 1 113 Stage 2 408 Stage 3	Combined region of right atrium, right coronary artery, and ascending aorta dose > 19.5 Gy associated with poorer survival	356 IMRT 805 3D-RT
Kim et al. 2022^18^	Single-center study of 560 patients with lung cancer treated with chemoradiotherapy 60–63 Gy in 1.8–2.1 Gy fractions	Substructures delineated by deep-learning–based autosegmentation and checked by radiation oncologists. SAN and AVN contoured manually by radiation oncologist	Overall survival Cardiac events	239 SCLC 321 NSCLC 51 Stages 1–2 509 Stage 3	Dmax to SAN ≥ 53.5 Gy associated with AF in patients with SCLC Dmax to SAN ≥ 20Gy associated with AF in patients with NSCLC Right atrium max dose associated with poorer survival in all patients	334 IMRT 226 3D-RT

3D-RT, three-dimensional radiotherapy; AF, atrial fibrillation; AVN, atrioventricular node; ECG, electrocardiogram; IBDM, image-based data-mining; IMRT, intensity-modulated radiotherapy; SABR, stereotactic ablative body radiotherapy; SAN, sinoatrial node; SVC, superior vena cava.

On the basis of this body of evidence, we have implemented a maximum dose limit to an anatomical region at risk at the base of the heart in all patients treated with curative-intent, non-stereotactic ablative body radiotherapy at our center.^6^^,^^7^ The dose limit is D1cc < 19.5 Gy in patients receiving 55 Gy in 20 fractions, D1cc < 21 Gy in patients receiving 60 Gy in 30 fractions and D1cc < 18.4 Gy in patients receiving 60 Gy in 15 fractions. To implement this dose limit, it is important that this new anatomical region at risk is consistently contoured; therefore, we developed an atlas to describe this anatomical region and an artificial intelligence auto-contouring solution to facilitate the implementation in the routine setting.^8^

## Translation From Data Mining to an Anatomical Region at Risk Contour

Using the significant *t*-level thresholds from IBDM, an anatomically relevant cardiac avoidance area (CAA) was developed by consensus opinion by a multidisciplinary team including a cardiologist with expertise in cardiac imaging (MS), two clinical oncologists with experience of treating lung cancer (KB, CFF), and the physicists who identified the anatomical region at risk region using IBDM (AMcW, MvH, AA).^2^, ^3^, ^4^

The CAA includes the SVC, right atrial appendage, right atrium (RA), aortic valve root, left main coronary artery (LMCA), proximal LAD, and proximal right coronary artery (RCA). The whole heart contour was defined as the pericardial sac, from the superior aspect of the pulmonary artery to the cardiac apex.^9^ The RA extends from SVC at the base of the heart and incorporates the sulcus terminalis, where the sinoatrial node (SAN) resides. The SAN is located at the junction of the RA and SVC and generates the cardiac impulse which spreads through anatomical routes in the RA to the atrioventricular node (AVN), located in the interatrial septum. The AVN delays the cardiac impulse before it is transmitted through the bundle of His and Purkinje fibers to ensure coordinated ventricular contraction. The coronary arteries supply the cardiac myocardium with blood and originate at the coronary ostia, immediately superior to the aortic valve.

Initially, five four-dimensional radiotherapy planning scans of the chest with contrast in patients with stage 1 lung cancer were selected from an ongoing clinical trial (NCT03645317). The SAN and AVN were contoured according to Loap et al.,^10^ and the LMCA, proximal portions of the LAD, and RCA were contoured according to the atlas by Duane et al.^11^ The aortic valve root was contoured to include the valve leaflets and their attachment to the aorta, which forms the aortic valve sinuses. Including the aortic sinuses ensured the inclusion of the origin of the right and left coronary arteries.

To facilitate the creation of an autocontouring solution for the CAA and its adoption in clinical practice, we included the LMCA and proximal LAD and RCA rather than the entirety of the coronary arteries. Autosegmentation of coronary arteries on radiotherapy planning CT images performs poorly with Dice similarity coefficients of 0.2 to 0.4.^12^ Proximal coronary artery occlusion is more likely to lead to acute coronary events compared with occlusion of distal coronary arteries and the RCA curves underneath the RA; therefore, it becomes included within the RA contour distally.

Loap et al.^10^ describe a cardiac contouring node delineation atlas using noncontrast simulation computed tomography (CT) scans from patients with breast cancer. Identifying the anatomical landmarks required to contour the SAN and AVN on four-dimensional CT planning scans proved challenging and would not be possible in routine clinical practice. Moreover, the substructures used as anatomical landmarks to contour the SAN and AVN move on respiration and cardiac contraction. Yan et al. evaluated the change in dose to cardiac substructures during the cardiac cycle and found that the absolute mean difference in dose to the RA myocardium and pulmonary artery is 3.1 Gy between end diastole and end systole.^13^ Consequently, we took a pragmatic decision to contour the whole of the RA according to Feng et al.^14^ to incorporate the SAN and AVN, to account for motion, achieve consistency and facilitate auto-contouring.

Once the constituent parts of the CAA had been agreed by consensus, they were contoured on the thorax window of the average intensity projection of four-dimensional CT scans with intravenous contrast from 10 patients with stage 3 lung cancer by one investigator (KB). The contours were reviewed by a cardiologist (MS). The constituent cardiac substructures were then combined to form the CAA.

## Description of a New Anatomical Region at Risk

**Figure 2:**
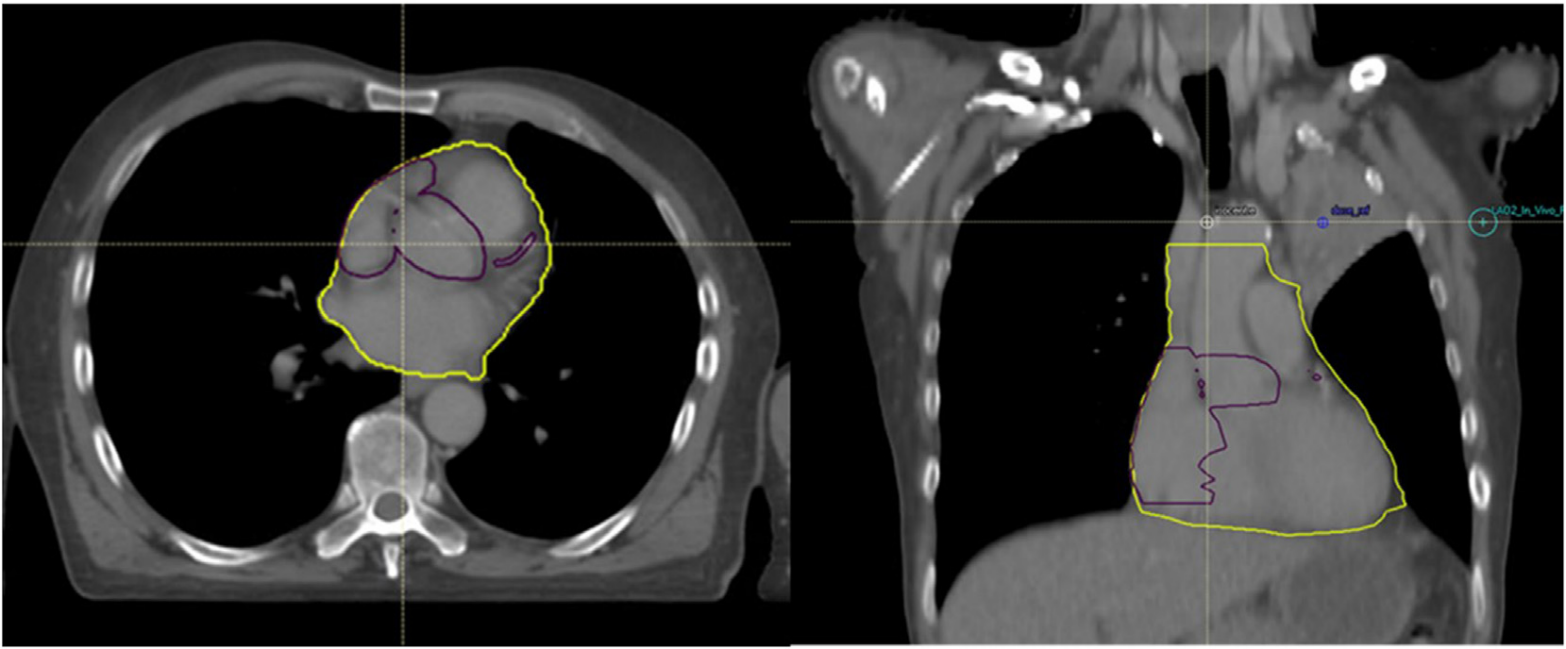
Cardiac avoidance area contour (purple) and heart contour (yellow) in axial and coronal views.

The CAA contour begins superiorly where the right atrial appendage becomes visible. It includes the right atrial appendage, the SVC, and the aortic valve root. An axial view is found in Figure 2. The right atrial appendage and SVC merge inferiorly to form the RA which is contoured as far as the inferior border of the heart. The inferior vena cava is excluded from the CAA. The aortic root includes the circumference of the aorta, extending from the coronary ostia superiorly to the atrioventricular septum inferiorly. The coronary arteries are contoured using a 5-mm rollerball. The LMCA is contoured from the aortic sinus on the left between the left atrium and pulmonary artery until it splits into the circumflex and LAD. The LAD is contoured from the end of the LMCA until it passes under the pulmonary artery. The RCA is contoured from the right aortic sinus to the heart border. A contouring atlas for the CAA can be found in the Supplementary Material. The boundaries of the CAA are described in Table 2.

**Table 2:** Boundaries of the Constituent Substructures of the Cardiac Avoidance Area

Substructure	Superior	Inferior	Lateral	Medial	Anterior	Posterior
Superior vena cava	Cranial axial image of the right atrial appendage	Caudal extent of SVC, where it joins the right atrium	Lateral edge of ascending aorta	Lateral border of the heart	Posterior edge of right atrium	Anterior edge of right pulmonary vein
Right atrium	Cranial axial image of the right atrial appendage	Caudal edge of the right atrium, at the cardiac apex	Lateral edge of aortic valve root and interventricular septum	Lateral border of the heart	Posterior border of the right ventricle, at the atrioventricular groove	Posterior border of the heart
Left main and left anterior descending coronary arteries	Cranial axial image of LMCA, at left aortic sinus	LAD passes beneath the pulmonary artery	Aortic sinus	2 cm from the left aortic sinus	5 mm rollerball to incorporate the diameter of the LMCA and LAD	
Right coronary artery	Cranial axial image of the RCA, at right aortic sinus	Lateral heart border	Aortic sinus	Right lateral border of the heart	5mm rollerball to incorporate the diameter of the RCA	
Aortic valve root	Cranial axial image at which right atrial appendage begins	Atrioventricular septum	Medial edge of the right atrium	Medial edge of left ventricle	Posterior edge of ventricles	Anterior edge of left atrium

LAD, left anterior descending coronary artery; LMCA, left main coronary artery; RCA, right coronary artery; SVC, superior vena cava.

## Adoption of a New Anatomical Region at Risk

Multiple studies have now revealed that thoracic radiotherapy in patients with lung cancer can cause cardiac related death and cardiac events; however, the exact mechanism of damage to the heart remains unclear. Consequently, we described the CAA, a new anatomical region at risk that incorporates critical structures which may be damaged by radiotherapy. Some of these structures, such as the coronary arteries and sino-atrial node, may have more serial than parallel features, and, therefore, a maximum dose threshold to the CAA is used as part of the base of the heart avoidance strategy.

Our data-mining work has led to the RAPID-RT research program that aims to use a rapid-learning methodology for the timely, safe, and evidence-based evaluation of changes to radiotherapy.^6^ Rapid-RT uses routinely collected data from electronic health records to assess outcome following the introduction of a dose limit to the CAA in patients undergoing curative-intent radiotherapy to the lung at our center. The maximum dose limit of 19.5 Gy for patients receiving 55Gy in 20 fractions was identified from IBDM as maximizing the survival difference. The dose limit is used in all patients having non-stereotactic ablative body radiotherapy and curative-intent radiotherapy for lung cancer and is adjusted, depending on the dose fractionation, for a biologically equivalent dose of 25.9 Gy, assuming an α/β for cardiac toxicity of 3.

The etiology of cardiac toxicity after lung cancer radiotherapy is complex, involving an interplay of preexisting cardiac comorbidities, cardiac risk factors, systemic therapy, and heart dose. All these variables should be evaluated through both real-world pragmatic studies and more conventional clinical trials to define the best treatment strategies for each individual patient. The coming years will reveal if the expected survival benefit of the base of the heart avoidance is realized in clinical practice.

## CRediT Authorship Contribution Statement

**Kathryn Banfill:** Conceptualization, Methodology, Formal analysis, Investigation, Writing – original draft, Visualization.

**Thomas Marchant:** Validation, Software, Data curation, Visualization, Writing – review and editing.

**Alan McWilliam:** Conceptualization, Software, Formal analysis, Writing – review and editing.

**Joseph Wood:** Software, Data curation, Writing – review and editing.

**Matthias Schmitt:** Methodology, Validation, Investigation, Writing – review and editing.

**Azadeh Abravan:** Software, Data curation, Writing – review and editing.

**Gareth Price:** Software, Data curation, Writing – review and editing.

**Marcel van Herk:** Supervision, Software.

**Corinne Faivre-Finn:** Conceptualization, Supervision.

## Disclosure

Dr. Banfill, Prof. van Herk, Prof. Faivre-Finn, Dr. Price and Dr. McWilliam have a patent pending WO2022018237A1 System and method for time-series imaging.
