# Supplementary material for: Brief Report of a New Anatomical Region at Risk in Thoracic Radiotherapy: From Discovery to Implementation
Source: JTO Clin Res Rep. 2024 Oct 18;5(12):100742. doi: 10.1016/j.jtocrr.2024.100742 (PMC11609655; doi:10.1016/j.jtocrr.2024.100742)
Supplement: Supplementary Material [file mmc1.pdf]

Cardiac Avoidance Area Contouring Atlas

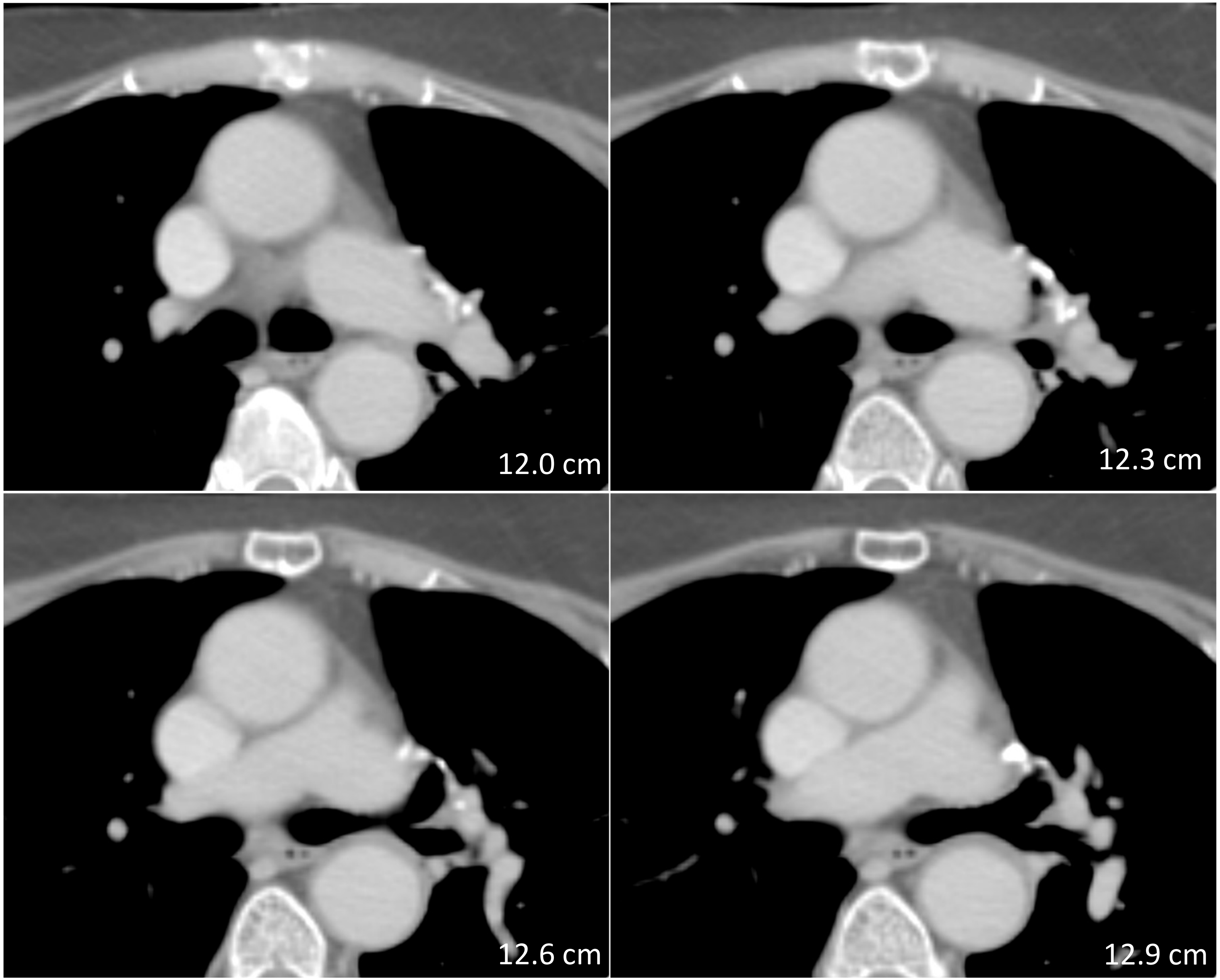
12.0 cm
12.3 cm
12.6 cm
12.9 cm

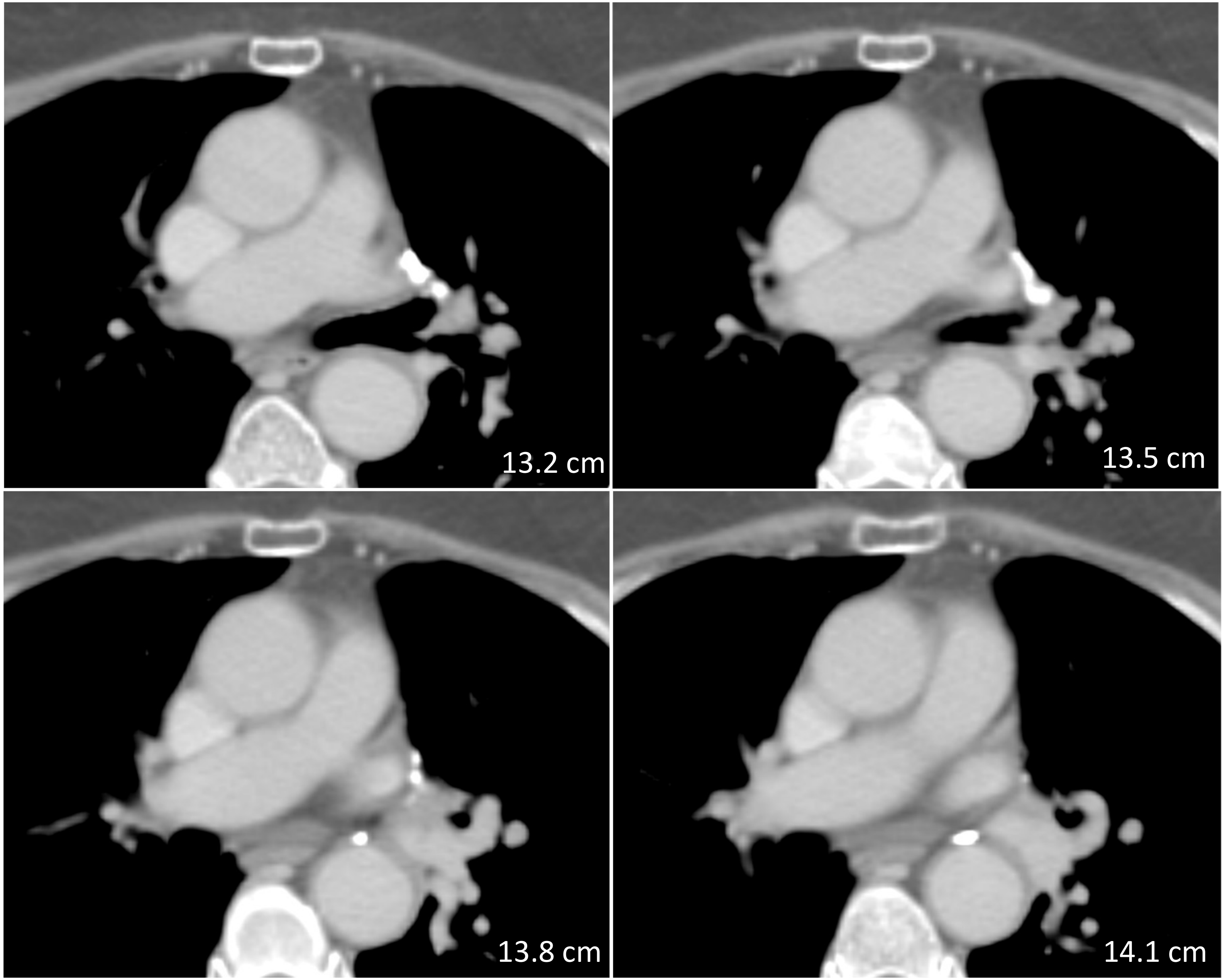
13.2 cm
13.5 cm
13.8 cm
14.1 cm

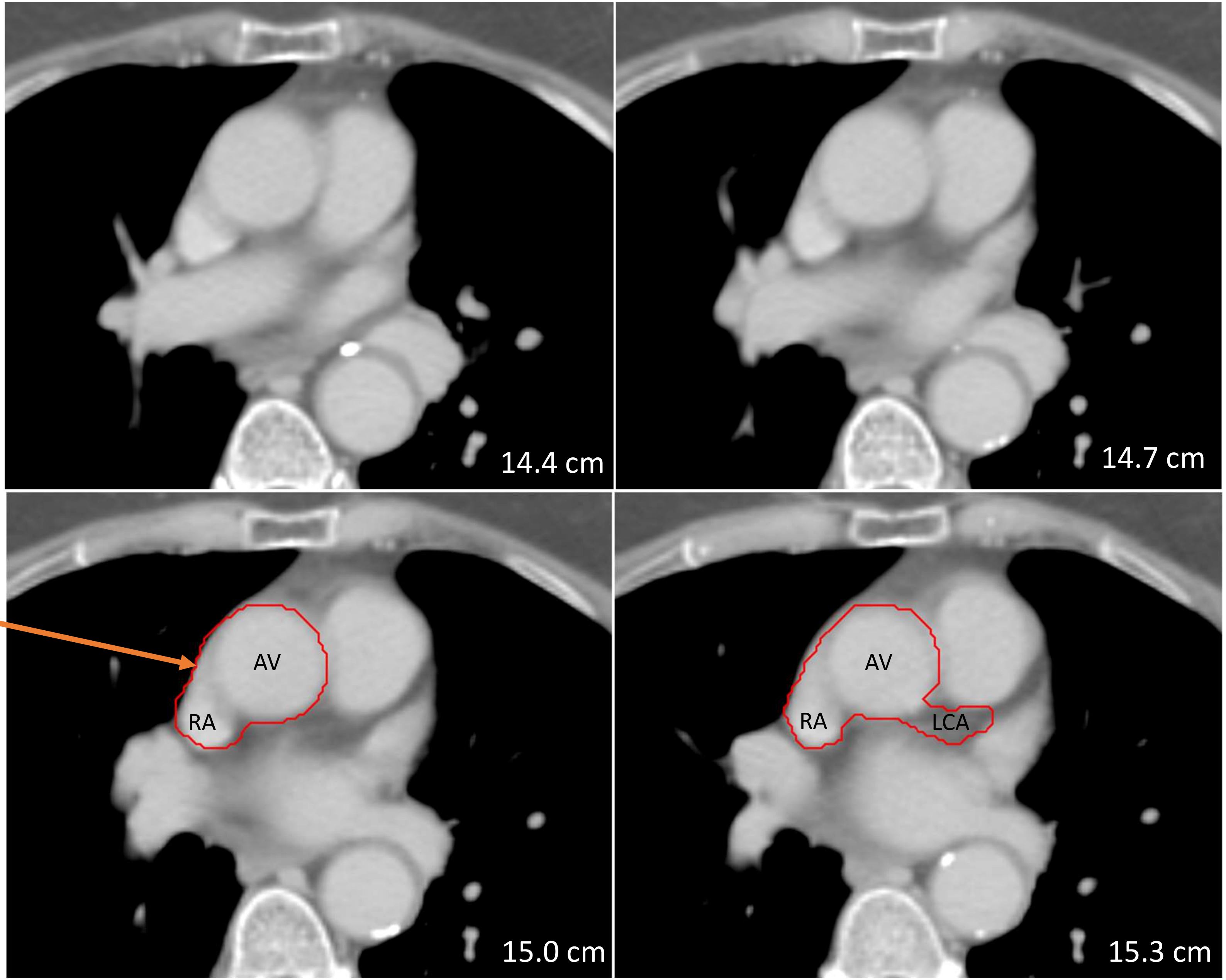

RA = Right Atrium

AV = Aortic Valve Root

LCA = L Coronary Artery

RCA = R Coronary Artery

Start contouring AV and RA when atrial appendage becomes visible

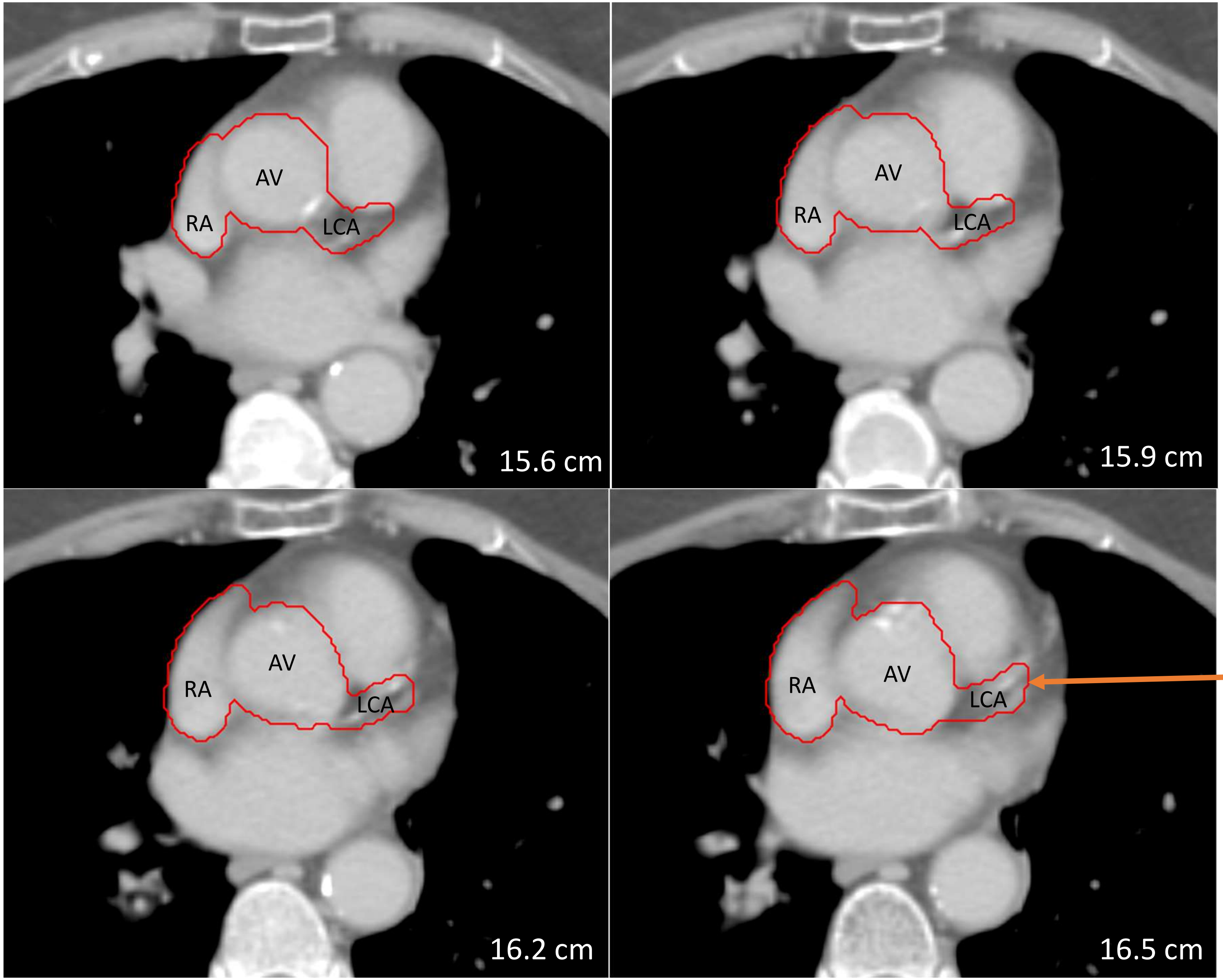

RA = Right Atrium

AV = Aortic Valve Root

LCA = L Coronary Artery

RCA = R Coronary Artery

Include LMCA and LAD up to approx 2cm from AV

L Circumflex not included

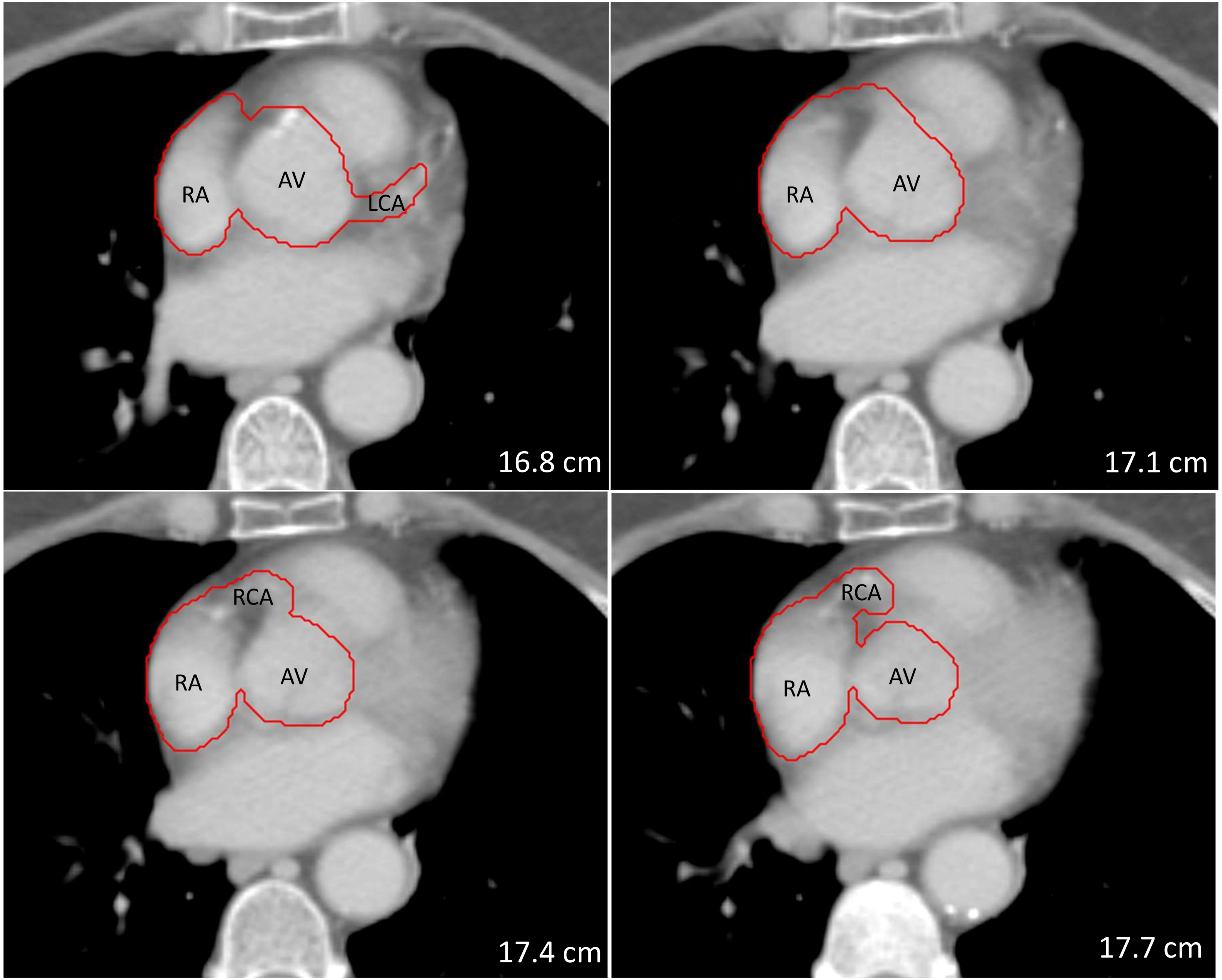

RA = Right Atrium

AV = Aortic Valve Root

LCA = L Coronary Artery

RCA = R Coronary Artery

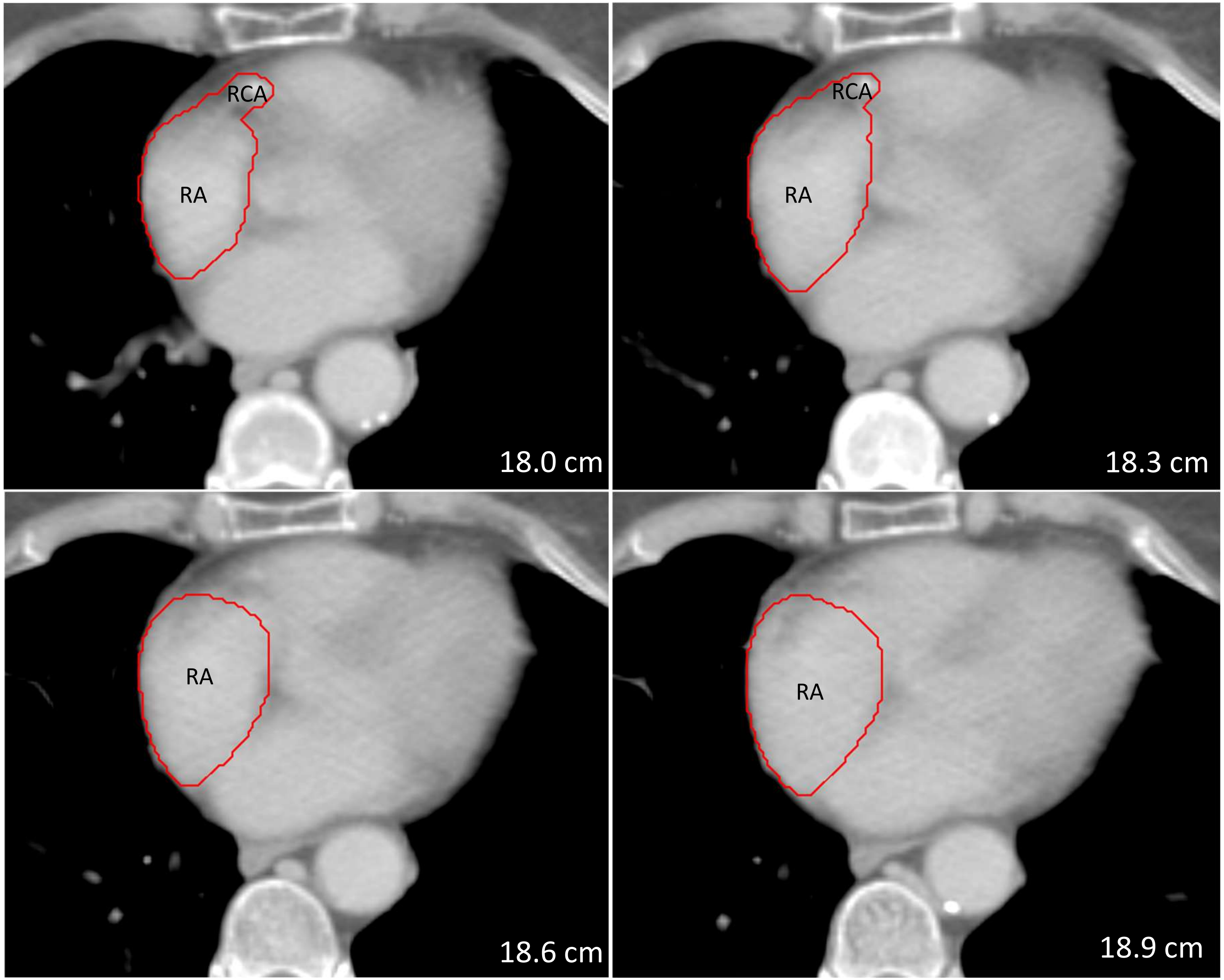

RA = Right Atrium

AV = Aortic Valve Root

LCA = L Coronary Artery

RCA = R Coronary Artery

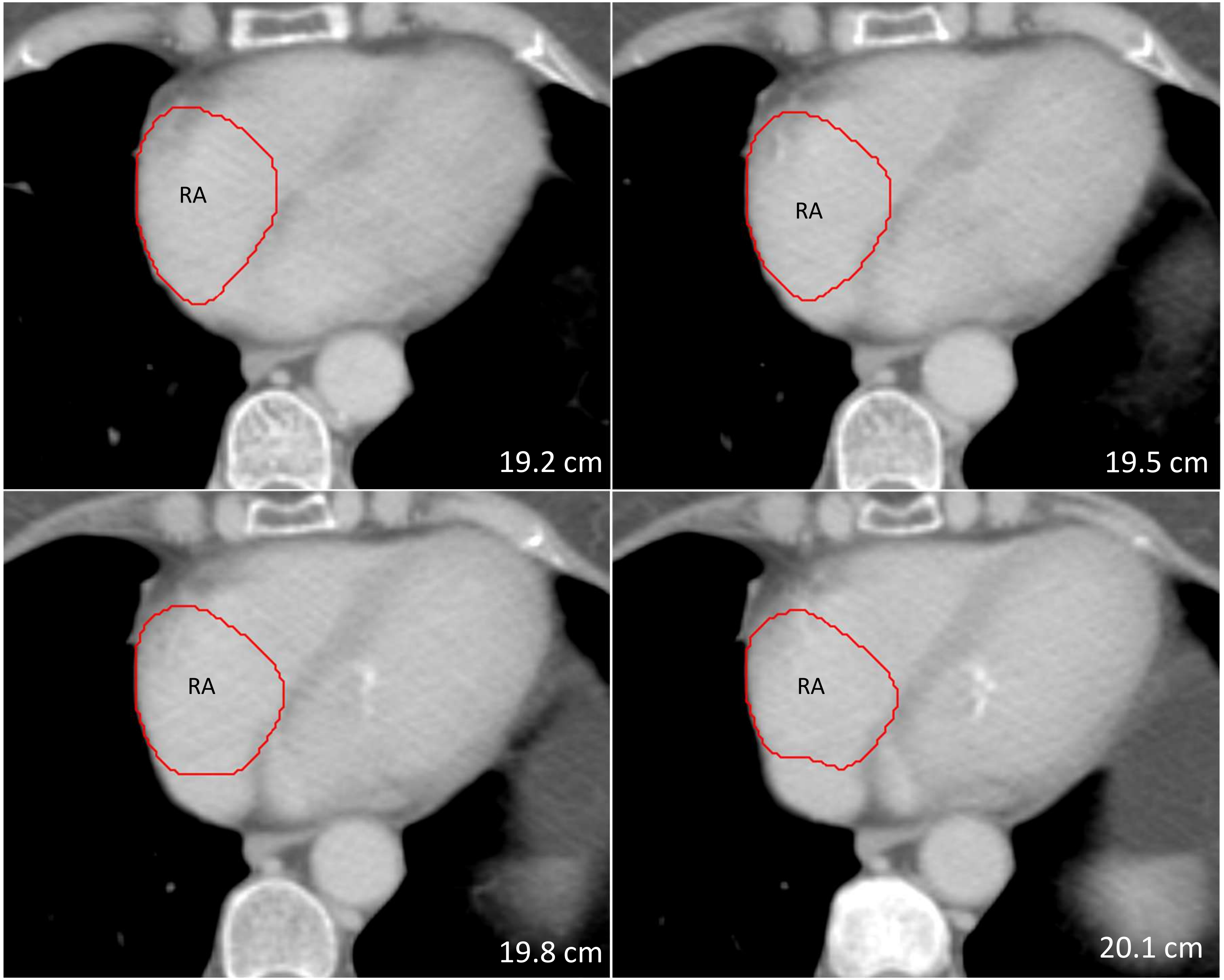

RA = Right Atrium

AV = Aortic Valve Root

LCA = L Coronary Artery

RCA = R Coronary Artery

Exclude Inferior Vena Cava from RA contour

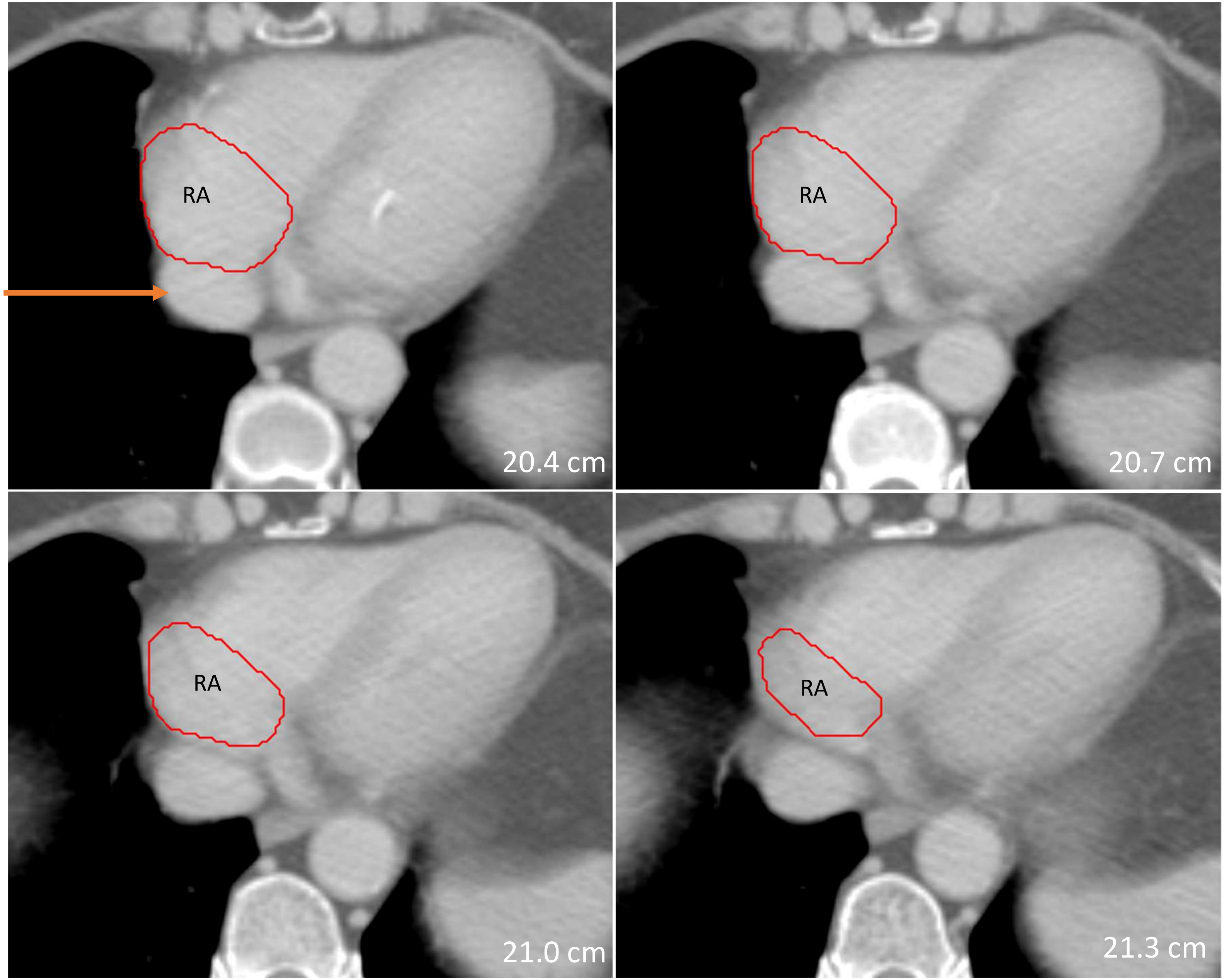

RA = Right Atrium

AV = Aortic Valve Root

LCA = L Coronary Artery

RCA = R Coronary Artery

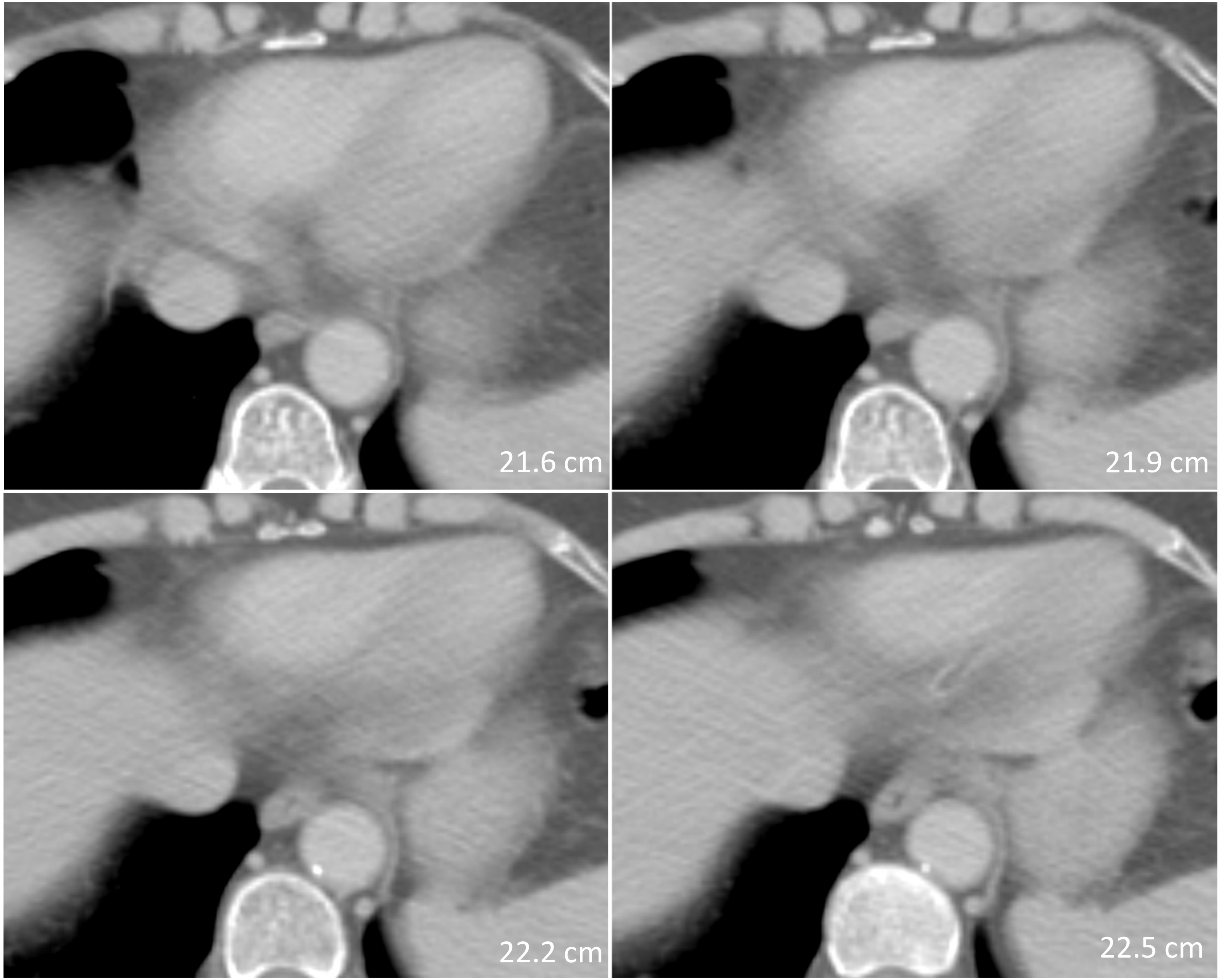
21.6 cm
21.9 cm
22.2 cm
22.5 cm

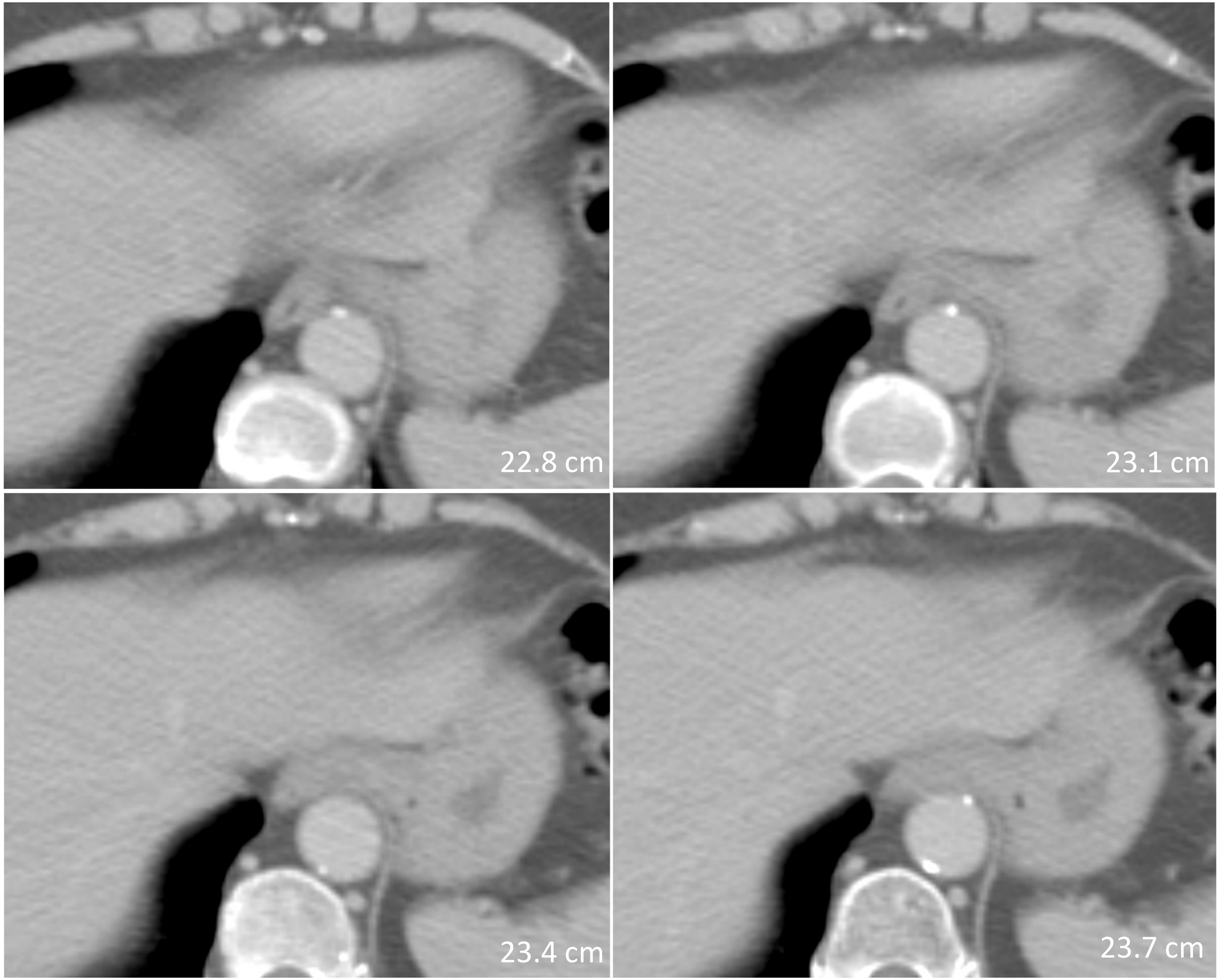
22.8 cm
23.1 cm
23.4 cm
23.7 cm

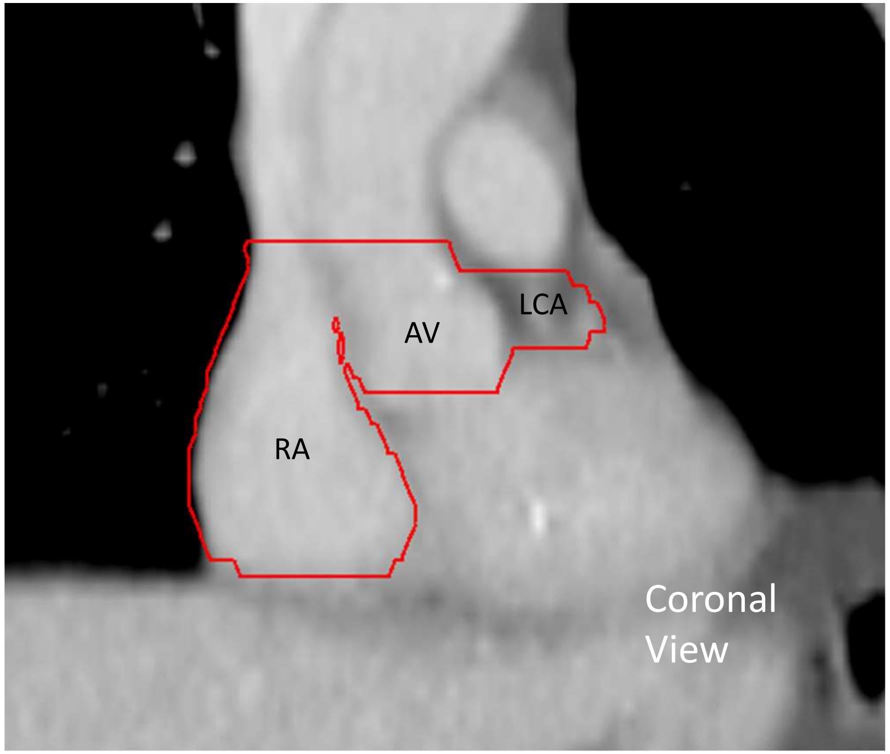

RA = Right Atrium

AV = Aortic Valve Root

LCA = L Coronary Artery

RCA = R Coronary Artery
